# A Systematic Review of Clinical Outcomes from Pharmacist Provided Medication Therapy Management (MTM) among Patients with Diabetes, Hypertension, or Dyslipidemia

**DOI:** 10.3390/healthcare10071207

**Published:** 2022-06-28

**Authors:** Srujitha Marupuru, Alexis Roether, A. J. Guimond, Chris Stanley, Tyler Pesqueira, David R. Axon

**Affiliations:** R. Ken Coit College of Pharmacy, The University of Arizona, Tucson, AZ 85721, USA; marupuru@pharmacy.arizona.edu (S.M.); aaroether@pharmacy.arizona.edu (A.R.); ajguimond@pharmacy.arizona.edu (A.J.G.); ctstanley@pharmacy.arizona.edu (C.S.); tjpesqueira@pharmacy.arizona.edu (T.P.)

**Keywords:** pharmacist, Medication therapy management, clinical outcomes

## Abstract

This study aimed to compare the clinical outcomes of pharmacist-provided medication therapy management (MTM) services as compared to no MTM services (i.e., standard of care) on outpatient clinical outcomes for patients with diabetes, hypertension, or dyslipidemia. A systematic literature review of PubMed, EMBASE, Cochrane library, International Pharmaceutical Abstracts, PsycINFO, Scopus, CINAHL electronic databases, grey literature, websites, and journals, was conducted from 1 January 2005–20 July 2021. The search field contained a combination of keywords and MeSH terms such as: “medication therapy management”, “pharmacist”, “treatment outcomes”. Studies published in United States, included adults ≥18 years old who received at least one pharmacist-provided MTM consultation and at least one group who received no MTM, and reported pre-specified clinical outcomes for diabetes mellitus, hypertension, or dyslipidemia were included. Of 849 studies identified, eight were included (cohort studies = 6, randomized controlled trials = 2). Clinical outcomes improved with MTM interventions, as evidenced by statistically significant changes in at least one of the three chronic conditions in most studies. Improvements were observed for diabetes outcomes (*n* = 4 studies), hypertension outcomes (*n* = 4 studies), and dyslipidemia outcomes (*n* = 3 studies). Overall, this study indicated that pharmacist delivered MTM services (versus no MTM services) can improve clinical outcomes for patients with diabetes, hypertension, and dyslipidemia.

## 1. Introduction

A core principle of quality patient-centered care is continual monitoring and evaluation to ensure that therapies are being utilized in an appropriate, safe, and effective manner [1]. In the United States (US), Medication Therapy Management (MTM) is a health service that can be performed by pharmacists to provide an informative and detailed review of a patient’s medication regimen. MTM was first outlined in The Medicare Prescription Drug, Improvement, and Modernization Act of 2003 [2]. Two common components of an MTM service are annual comprehensive medication reviews (CMRs) and quarterly targeted medication review (TMRs) [3,4]. In 2006, the Centers for Medicare and Medicaid Services (CMS) established the Medicare Part D program that requires MTM services to be offered to eligible beneficiaries [5].

The main goals of MTM services are the prevention of adverse drug events (ADEs), improved medication adherence, and patient education leading to appropriate medication use [2]. Since its inception, MTM has evolved from being directed toward acute medication education to helping manage medications for chronic conditions and the costs associated with them [6]. Previous studies on MTM interventions are consistent with the idea that positive outcomes are attributed to patient interaction about their medication regimen. Such studies have found that MTM leads to lower hospital readmission, healthcare costs, medication issues, and improved medication adherence [6,7,8,9].

However, there is a need for evidence-based information on the effectiveness of MTM on the three most common chronic conditions in the US: type 2 diabetes mellitus, hypertension, and dyslipidemia.

Diabetes is highly prevalent in the US, affecting at least 30.3 million people or around 9.4% of the population [10]. Many medications for diabetes are effective at controlling blood glucose and HbA1c levels but their effect can be inadequate if not taken correctly. Pharmacists have a role in helping patients who take diabetes medications achieve their target goals, typically defined by the American Diabetic Association (ADA) as maintaining blood glucose levels between 70-130 mg/dL and HbA1c less than 7% [11,12].

Hypertension is defined as blood pressure ≥130/80 mmHg and affects almost half of US adults (116 million), of which a majority do not have their blood pressure under control (92.1 million) [13]. Hypertension also adversely affects the kidneys, brain, and arterial blood vessels leading to major comorbidities and complications [14]. Lifestyle changes can help lower blood pressure to an extent before pharmacological treatment has to be utilized.

Dyslipidemia is considered an imbalance in total cholesterol, low-density lipoprotein (LDL), high-density lipoprotein (HDL), and triglycerides [15]. Dyslipidemia is more prevalent in adults over the age of 20, with 11.5% of US adults having a high serum total cholesterol (≥240 mg/dL) in 2018 [16].

Pharmacists are experts in medications and therefore have an important role helping patients to optimize their medication regimens for these three common conditions through MTM services. Pharmacists are also trained to assess and determine the appropriateness of medications, alleviate barriers to adherence, and provide education regarding the medications, such as common adverse drug events (ADEs) and guidance on correct administration [17]. Pharmacists therefore have an important role in MTM to identify and find solutions for various medication-related problems, including drug-drug and drug-disease interactions and monitoring for adjustments that need to be made such as renal and hepatic impairment.

The objective of this systematic review was to assess the impact of pharmacist provided MTM interventions when compared to no intervention on pre-defined clinical outcomes among outpatients with type 2 diabetes mellitus, hypertension, or dyslipidemia.

## 2. Methods

### 2.1. Search Strategy

**Figure 1:**
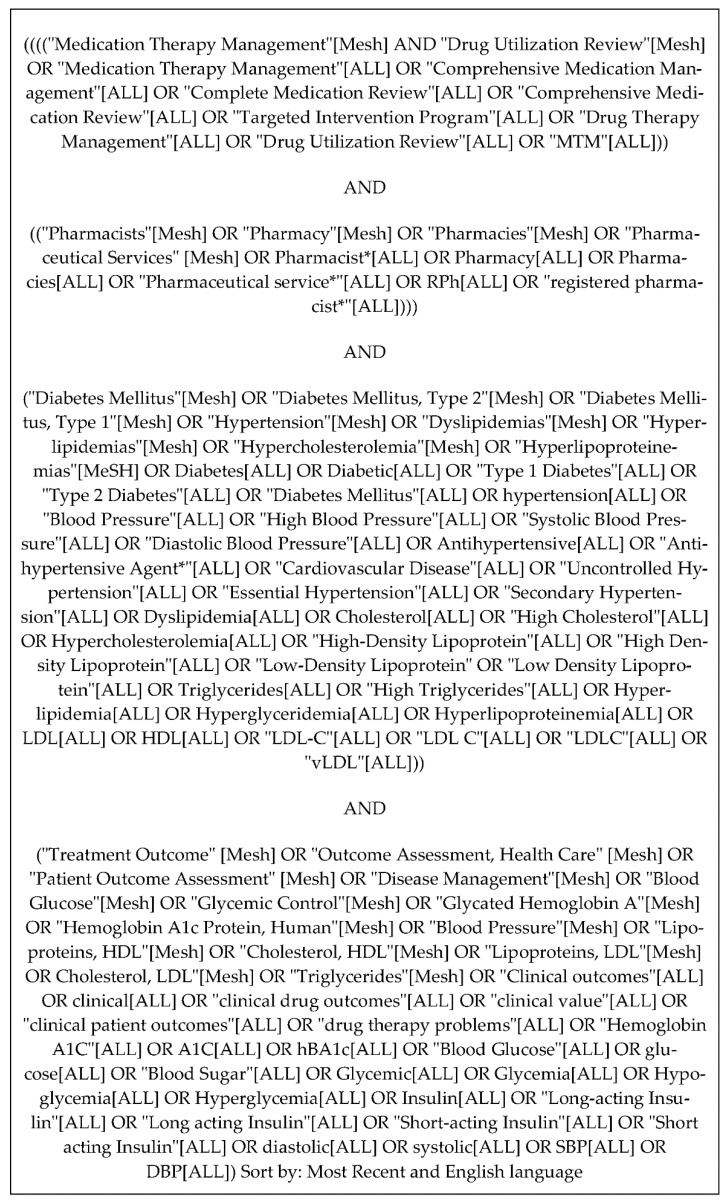
Medline/PubMed search strategy.

This systematic review followed the Preferred Reporting Items for Systematic Reviews and Meta-Analyses (PRISMA) criteria [18]. A focused systematic literature search strategy was developed by the research team to identify relevant articles. The search strategy used a combination of medical subject headings (MeSH) and title and abstract keywords. The search strategy was adapted for use in seven electronic databases: PubMed/Medline (NLM), Embase, Cochrane Library, Scopus, PsycINFO (Ebsco), Cumulative Index to Nursing and Allied Health Literature (CINAHL) and the International Pharmaceutical Abstracts (IPA). A grey literature search was also conducted, which included ClinicalTrials.gov and OAIster. Furthermore, the reference lists of identified articles were screened to identify additional studies. The following keywords were used to search the databases and grey literature: medication therapy management, pharmacists, diabetes mellitus, hypertension, dyslipidemia, and treatment outcome. Additionally, keyword synonyms and other search terms were used to ensure the search was as comprehensive as possible. The search strategy was developed in PubMed (Figure 1) and adapted for the other databases.

### 2.2. Eligibility Criteria

To be included in this systematic review, the study had to compare at least one episode of pharmacist provided MTM service (intervention group) via any mode of delivery (e.g., telephonic or in-person) to standard care without MTM services (comparison group), include patients with diagnosis of diabetes, hypertension and/or dyslipidemia, report relevant clinical outcomes, (e.g., change in systolic blood pressure [SBP] or diastolic blood pressure [DBP], percentage meeting goal of blood pressure <130/80 mm of mercury [mmHg], Hemoglobin (Hb) A1c levels, percentage meeting goal of HbA1c <7%, change in low-density lipoprotein (LDL) and high-density lipoprotein (HDL) values, percentage meeting goals of LDL <100 mg/dl total cholesterol level), include patients aged ≥18 years old, written in English, set in the US, be considered primary research (e.g., randomized control trial (RCT), cohort, survey/questionnaire, interview, case-control), and published between 1 January 2005 (this date was chosen because the concept of MTM was first implemented in 2005) and 20 July 2021 (when the search was conducted).

### 2.3. Study Selection

The titles and abstracts of identified articles were screened by at least two independent reviewers (A.R., A.G., or C.S.) to determine eligibility for inclusion using a screening tool developed specifically for this study. This tool screened for the following items: if the study is primary research, published between 1 January 2005, and 20 July 2021, conducted in the US and published in English, has an intervention group that received at least one episode of an MTM service, and reports outcomes related to diabetes, hypertension, or dyslipidemia. Abstracts that were unclear if they met the inclusion criteria were included for full text review to determine eligibility. Then, at least two independent reviewers (A.R., A.G., or C.S.) assessed the full text of articles that were identified as potentially relevant in the screening process and extracted relevant data using a data extraction tool that was also developed specifically for this study. The data extraction tool included the following items: patient age, gender, number of patients in MTM intervention and no MTM group, number of MTM services completed, length of time for MTM intervention, study settings, insurance status, type of chronic conditions, characteristics of the MTM services provided, characteristics of no MTM or standard care, and MTM outcomes as reported in the studies for MTM services and No MTM group. In instances where it was unclear whether the article should be included, a discussion was held with other research team members (D.R.A. and S.M.) until consensus was reached. EndNote (Version 20, Clarivate Analytics, Philadelphia, PA, USA) and Mendeley (Version 1.19.6) were used for citation management. Data were compiled using Microsoft Excel (Version 16.48, Microsoft, Redmond, WA, USA).

### 2.4. Risk of Bias Assessment

Two independent reviewers (S.M. and T.P.) assessed risk of bias in the included studies. Risk of bias in randomized controlled trials was assessed using the Cochrane Risk of Bias tool for RCTs (RoB 2) [19]. This tool assessed six bias domains: (1) selection; (2) performance; (3) detection; (4) attrition; (5) reporting; and (6) other bias, which could be reported as having low risk, some concerns, or high risk of bias [19]. Risk of bias in observational studies was assessed using the Cochrane Risk of Bias in Non-Randomized Studies of Interventions (ROBINS-I) tool [20]. This tool has seven bias domains: (1) confounding; (2) selection of participants into the study; (3) classification of interventions (4) deviations from intended interventions; (5) missing data; (6) measurement of outcomes; and (7) selection of the reported result, which could be reported as having a low, moderate, serious, or critical risk of bias [20]. A third independent reviewer (D.R.A.) was consulted as needed to resolve any differences between the two initial reviewers.

## 3. Results

### 3.1. Study Selection

**Figure 2:**
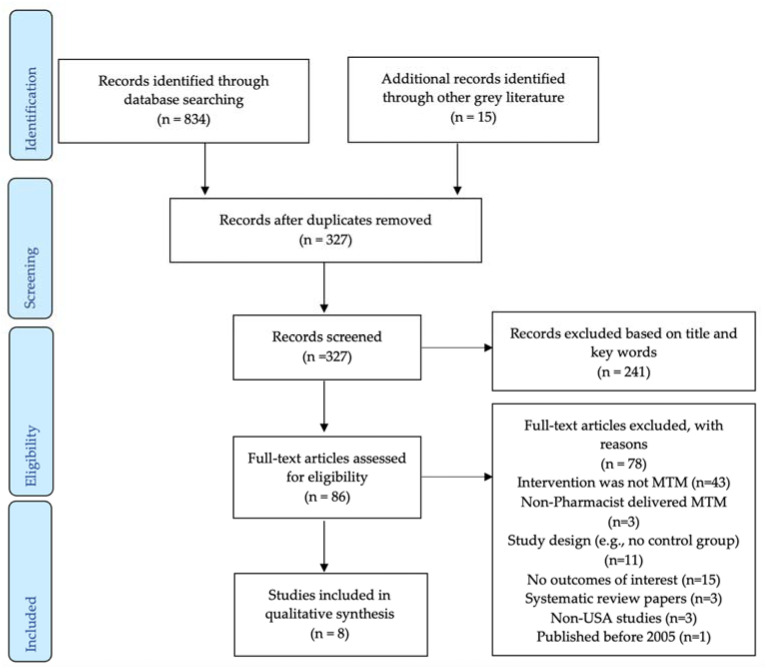
PRISMA flowchart.

The study selection process is described in Figure 2. There were 327 unique records that were screened after duplicates were removed, of which 86 articles underwent full-text review. A total of eight articles were ultimately included in the systematic review [21,22,23,24,25,26,27,28].

### 3.2. Characteristics of Included Studies

Table 1 displays the characteristics of the eight included studies, which included two RCTs [21,22] and six cohort studies [23,24,25,26,27,28]. Four studies included patients with diabetes [23,24,26,27], three studies included patients with diabetes and/or hypertension [21,22,28], while one further study included patients diagnosed with selected chronic conditions among which diabetes, hypertension and dyslipidemia were included [25]. The total number of study subjects ranged from 52 [22] to 2681 [25].

### 3.3. Results of Individual Studies

Table 2 presents the clinical outcomes reported in included studies. The findings are summarized here by diabetes, hypertension, and dyslipidemia outcomes.

Two studies reported the percentage of people who had HbA1c <7% [23] or <8% [26] while another study reported the percent change in the number of people with HbA1c <7% [25]. Two studies reported mean HbA1c values [27,28].

Two studies reported the percentage of people who had BP <130/80 mmHg [22,23] or BP <140/80 mmHg [26]. Four studies reported mean SBP [22,27,28] or mean change in SBP [21] while three studies reported mean DBP [27,28] or mean change in DBP [21].

Two studies reported the percentage of people who had LDL <100 mg/dL [23,24] while another study reported the percent change in the number of people with LDL <100 mg/dL [25]. Three studies reported the mean LDL value [24,27] or mean change in LDL values [21] while two studies reported the mean HDL value [27] or mean change in HDL values [21]. One study reported the percentage of people who had a statin prescription [26].

### 3.4. Risk of Bias in Included Studies

Findings from the risk of bias assessment are presented in Table 3. The overall risk of bias was considered low in one RCT [22] and moderate in the other RCT [21], while the overall risk of bias was considered moderate for all six cohort studies [23,24,25,26,27,28].

## 4. Discussion

The findings from this systematic review add to the growing body of literature that describes the potential benefits of pharmacist provided MTM services for some of the most common chronic conditions in the US. The evidence basis for this review consisted of eight studies (two RCTs trials and six observational studies). The two RCTs compared an MTM services intervention with usual care or control rather than with a different active intervention whereas most observational studies were cohort studies. This systematic review found that current evidence from the included studies suggests that pharmacist provided MTM services have statistically significant results showing improved clinical outcomes in at least one of the three conditions: diabetes, hypertension, and dyslipidemia.

Four of the six included studies observing blood pressure as a primary clinical outcome showed decreased systolic and diastolic blood pressure readings or a greater percentage of people reaching the blood pressure goal of <130/80 mmHg after the patient received MTM from a pharmacist [21,22,26,28]. In four studies looking at the clinical outcomes of diabetes, the percentage of patients with HbA1C <7% was higher, and mean HbA1c values reduced, after receiving pharmacist led MTM services [23,26,27,28]. For dyslipidemia, two studies showed a statistically significant reduction in LDL levels and higher percentage with statin prescription after patients received MTM services from a pharmacist [24,26]. Two studies also observed a change in HDL as a clinical outcome and showed an increase in HDL levels, although the changes were not statistically significant (*p* > 0.05) [21,27]. The findings for each of these three conditions is discussed in greater detail below.

### 4.1. Diabetes

In the four studies that showed improved biomarkers of diabetes, different objective measurements were used. Two studies reported significant mean HbA1c improvement during the study period [27,28] while the other two focused on measuring the percentage of patients achieving the HbA1c goal of <7% or 8% in a year study period [23,26]. The therapeutic goals were based on the clinical practice guidelines that were set in studies such as D5 diabetic measure by Minnesota Community Measurement (MNCM) and 2013 ACC/AHA guideline, respectively. The D5 diabetic measure consists of five treatment goals set by MNCM for diabetic patients; blood pressure control, lowering bad cholesterol, blood sugar maintenance, avoid tobacco and as take aspirin as recommended [29]. One further study that looked at the percentage of diabetic patients having HbA1c <7% showed a positive trend towards better diabetic outcomes among those who declined MTM intervention. This change was not statistically significant and was based on 6-months outcome analysis [25]. One reason to explain this finding could be that patients who declined MTM may have had this chronic disease for many years and are actively engaged in the self-management of their diabetes. Thus, additional education and disease monitoring is not a priority for them. A previous randomized controlled trial looking at pharmaceutical care program with clinical pharmacists’ intervention versus a control group with outpatient care among patients with diabetes also found no significant changes in mean HbA1c [30]. Clifford et al. concluded that a longer time frame and increased intensity of intervention may be required to show a clinical improvement in glycemic control. Nevertheless, MTM is an important part of managing diabetes since pharmacists providing patient education, monitoring therapy goals, and making appropriate interventions has demonstrated better control of diabetes [31,32].

### 4.2. Hypertension

Four of the six studies in our review showed a statistically significant improvement in objectively measured reduction in blood pressure, and they varied in the measurement parameters. Two studies used mean change in SBP and DBP values [21,28] while one looked at the percentage change in patients achieving hypertensive goal of <130/80 mmHg [22] and another looked at the percentage who achieved <140/90 mmHg [26]. Two studies reported no significant change in hypertensive outcomes, and they attributed this to the relatively small sample size in their studies [23,27]. These results are similar to those reported in another systematic review [33] demonstrating a positive association between pharmacist interventions among hypertension patients. This review consisted of 35 studies among which 29 of them showed statistically significant improvement in blood pressure in the intervention group at follow-up. However, this systematic review included only randomized control trials and any pharmacist-led intervention with medication counseling, patient education, distribution of interventions materials, individualized care plans, and check-in meetings [33].

### 4.3. Dyslipidemia

Among the six studies looking at LDL and HDL clinical outcomes among dyslipidemia patients, only two of them showed significant improvements compared to the no-MTM group [24,26]. Pharmacist provided MTM services increased the percentage of patients attaining the LDL goal of <100 mg/dL [24] and found a significant change in the statin prescription between patients seen by the pharmacist in MTM setup and the usual care [26]. Similar to our findings, previous work has concluded that overall, there is insufficient evidence to support the effectiveness of MTM interventions on lowering mean LDL-cholesterol levels or increasing the percentage of patients achieving an LDL-cholesterol goal [9]. The authors commented that findings were imprecise, and the magnitude of effect was inconsistent with the RCTs and observational study findings [9].

### 4.4. Clinical Implications

The results of this systematic review provide evidence that pharmacist provided MTM services have a role in improving clinical outcomes in the outpatient setting. Pharmacists are uniquely and ideally positioned to provide various services such as patient education, quality follow-up, monitoring and encouraging health-promoting behavior needed for improved clinical outcomes [34,35]. With pharmacists being the most accessible members of a healthcare team [36], pharmacist provided MTM services provides an opportunity to help patients especially high-risk and vulnerable population. MTM services take a more proactive approach to a patient’s healthcare and has broad applications in community-based settings. Given that diabetes, hypertension, and dyslipidemia are three of the most common chronic conditions in the US, such MTM services targeted to these populations is warranted to help improve health outcomes.

### 4.5. Limitations

There were some limitations to this systematic review. Although MTM services must meet certain standards, MTM providers have some flexibility in how services are provided. Therefore, there may be some variation in how MTM services were administered depending on the site administering them. The heterogeneity of MTM services means findings within this review must be interpreted with caution as any potential differences may impact the findings. Furthermore, wide variations in the MTM interventions may have led to some articles being excluded due to the pre-defined inclusion criteria and the varied terminologies. This review focused only on the adult population with three of the most common chronic conditions (diabetes, hypertension, and dyslipidemia). Each of these conditions may be defined differently depending on the guidelines or definitions used in the individual study, which may influence the results. Our review did not address MTM interventions conducted in inpatient settings or single-episode types of interventions (e.g., medication reconciliation, which some view as a specific type of MTM service). Our evidence base collected in this study did not include the interventions labeled pharmaceutical care or medicines management or community pharmacy health management program and we strictly ensured that an MTM descriptor was present to reduce heterogeneity. We stringently excluded any studies that were of disease or case management interventions, which could explain the small number of studies included in our review. This review only included studies from the US, given that pharmaceutical care is defined and practiced differently in other countries. Thus, our findings are not generalizable beyond the eligibility criteria of our review.

### 4.6. Future Directions

Further studies with longer follow-up periods are needed to provide more definitive evidence that pharmacist provided MTMs can improve objective clinical biomarker outcomes among patients with diabetes, hypertension, and/or dyslipidemia. We hypothesize that more studies will allow researchers to conduct a meta-analysis on pharmacist delivered MTM services and assess how it affects outcomes relative to patient disease states in future. In addition, given that these conditions are complex with several factors influencing their outcomes [37,38], further studies are warranted with different intensity of interventions, populations, and settings [39,40,41].

## 5. Conclusions

The findings of this systematic review indicate that pharmacist delivered MTM services can improve clinical outcomes for patients with diabetes, hypertension, and dyslipidemia, when compared to no MTM services. These findings lend further evidence to the value of pharmacist provided MTM services for patients with common chronic conditions. Future research could be conducted to help provide more targeted and definitive evidence for the value of this service.

## Figures and Tables

**Table 1:** Characteristics of included studies.

Author, Year	Study Design	Settings	Insurance Status	Eligible Conditions	Mode of Delivery	Team	Frequency of Follow Up/Year	Total *N*	Mean Age Patients	% Male Patients
Hirsch, 2014 [21]	RCT	University-based primary care clinic	All plans	DM, HTN	Face to face and telephone	Collaborative pharmacist–primary care provider	≥4	667	67.5	42.6
Planas, 2009 [22]	RCT	Community pharmacy	Insured	DM, HTN	Face to face	Pharmacist	12	52	64.7	37.2
Brummel, 2006 [23]	Cohort	Comprehensive provider of pharmacy service	Medicare, Medicaid	DM	Face to face	Pharmacist	Any	224	58.3	48.2
Fox, 2009 [24]	Cohort	Managed care organization (Florida Health Care Plans)	Medicare D	DM	Telephone	Collaborative pharmacist–primary care provider	≥3	2114	69.2	50.1
Pindolia, 2009 [25]	Cohort	Health Alliance Plan	Health alliance plan	26 possible chronic conditions *	Telephone	Collaborative pharmacist–primary care provider	N/A	2681	73.7	39.8
Prudencio, 2018 [26]	Cohort	Patient-Centered Medical Home	N/A	DM	Face to face	Pharmacist	≥1	811	63.0	49.0
Skinner, 2015 [27]	Cohort	Community health center	N/A	DM	Face to face	Pharmacist	≥4	58	53.7	42.0
Tilton, 2019 [28]	Cohort	Academic health center	Low income	DM, HTN	Face to face	Pharmacist	≥3	316	69.6	40.5

RCT = randomized controlled trial; N/A = data not available; DM = diabetes mellitus; HTN = hypertension. * 26 possible chronic diseases included Alzheimer’s disease, ankylosing spondylitis, arthritis, asthma, Benign prostatic hyperplasia, Coronary artery disease, Congestive heart failure, cancer, chronic obstructive pulmonary disease, depression, diabetes, epilepsy, erectile dysfunction, Fabry’s disease, hepatitis B/C, HIV/AIDS, hypertension, insomnia, multiple sclerosis, obesity, osteoporosis, Parkinson’s disease, plaque psoriasis, stress/urge incontinence, stroke/transient ischemic attack.

**Table 2:** Clinical outcomes reported in included studies.

Author, Year	N Analyzed in Each Group	Follow-Up Period	Outcomes Reported	Intervention	Control	*p* Value
Hirsch, 2014 [21]	I-75 C-91	Baseline, 3, 6, and 9 months	Mean ± SD change in LDL (mg/dL)	6 months: 0.1 ± 19.9	6 months: 4.6 ± 24.1	0.21
				9 months: −3.5 ± 26.3	9 months: −3.1 ± 41.9	0.95
			Mean ± SD change in HDL (mg/dL)	6 months: 2.4 ± 28.3	6 months: 0.3 ± 11.5	0.54
				9 months: −1.0 ± 20.4	9 months: 0.4 ± 20.9	0.67
			Mean ± SD change in SBP (mmHg)	6 months: −7.1 ± 19.4	6 months: 1.6 ± 21.0	**0.008**
				9 months: −5.2 ± 16.9	9 months: −1.7 ± 17.7	0.22
			Mean ± SD change in DBP (mmHg)	6 months: −3.8 ± 10.5	6 months: 1.7 ± 13.9	**0.006**
				9 months: −2.5 ± 10.2	9 months: −0.3 ± 13.8	0.27
Planas, 2009 [22]	I-32 C-20	Monthly within study period of 9 months	% With BP <130/80 mmHg at 9 months	48.00	6.67	**0.021**
			Mean SBP (mmHg) at 9 months	124.44	148.13	**0.003**
Brummel, 2013 [23]	I-121 C-103	2006, 2007, 2008	% With HbA1c <7%	2006: 43.80	2006: 63.11	**0.003**
				2007: 73.55	2007: 72.82	0.90
				2008: 42.15	2008: 59.22	**0.01**
			% With LDL <100 mg/dL	2006: 63.64	2006: 65.05	0.82
				2007: 83.47	2007: 73.79	0.07
				2008: 79.34	2008: 73.79	0.32
			% With BP <130/80 mmHg	2006: 66.12	2006: 61.17	0.44
				2007: 71.07	2007: 72.82	0.77
				2008: 76.03	2008: 69.90	0.30
Fox, 2009 [24]	I-255 C-56	1 January 2006– 30 September 2007	% With LDL <100 mg/dL	69.00	50.00	**<0.001**
			Mean ± SD LDL (mg/dL)	83.4 ± 31.2	90.8 ± 31.0	**<0.001**
Pindolia, 2009 [25]	I-520 C-2161	2006, 2007	% Change in people with HbA1c <7%	3	7	N/A
			% Change in people with LDL <100 mg/dL	−5	7	N/A
Prudencio, 2018 [26]	I-95 C-132	1 October 2014– 31 October 2015	% With HbA1c <8%	54	36	**0.010**
			% With BP <140/90 mmHg	93	77	**0.001**
			% With statin prescription	79	63	**0.010**
Skinner, 2015 [27]	I-50 C-50	12-month	Mean ± SD HbA1c (%)	7.5 ± 0.38	10.8 ± 2.0	**<0.01**
			Mean ± SD LDL (mg/dL)	92.7 ± 36.4	110.8 ± 65.7	0.17
			Mean ± SD HDL (mg/dL)	48.2 ± 10.3	45.2 ± 12.9	0.16
			Mean ± SD SBP (mmHg)	136.5 ± 19.8	145.4 ± 17.8	0.12
			Mean ± SD DBP (mmHg)	72.7 ± 10.3	73.8 ± 14.7	0.63
Tilton, 2019 [28]	I-158 C-158	2001–2011	Mean ± SD HbA1c (%)	6 months: 7.39	6 months: 7.56	**0.007**
				12 months: 7.49	12 months: 7.75	**0.016**
			Mean ± SD SBP (mmHg)	6 months: 135.3	6 months: 135.2	**0.011**
				12 months: 133.0	12 months: 134.6	**0.002**
			Mean ± SD DBP (mmHg)	6 months: 72.8	6 months: 76.3	**0.014**
				12 months: 72.2	12 months: 73.6	0.269

I = intervention; C = comparator; SD = standard deviation; LDL = low density lipoprotein; mg/dL = milligrams per deciliter; HDL = high density lipoprotein; SBP = systolic blood pressure; mmHg = millimeters of mercury; DBP = diastolic blood pressure; BP = blood pressure; HbA1c = hemoglobin A1c; DM = diabetes mellitus; N/A = data not available. Bold indicates significant *p*-value. The “negative sign” in front of lab values indicate the change in the clinical outcome. Tilton, 2019 study has missing standard deviations in their clinical outcomes reported.

**Table 3:** Risk of bias assessment in included studies.

Randomized Controlled Trials								
Author, Year	Randomization	Deviations from Intended Intervention	Missing Outcome Data	Measurement of Outcome	Selection of Reported Results	Overall		
Hirsch, 2014 [21]	Low	Low	Some Concerns	Low	Low	Moderate		
Planas, 2009 [22]	Low	Low	Low	Low	Low	Low		
**Cohort Studies**								
**Author, Year**	**Confounding**	**Selection of** **Participants**	**Classifications** **of Interventions**	**Deviations of** **Interventions**	**Missing Data**	**Measurement** **of Outcomes**	**Selection of** **Reported Results**	**Overall**
Brummel, 2013 [23]	Moderate	Low	Low	Moderate	Moderate	Moderate	Low	Moderate
Fox, 2009 [24]	Moderate	Low	Low	Low	Moderate	Low	Low	Moderate
Pindolia, 2009 [25]	Moderate	Moderate	Moderate	Low	Low	Low	Low	Moderate
Prudencio, 2018 [26]	Moderate	Low	Low	Low	Low	Low	Low	Moderate
Skinner, 2015 [27]	Moderate	Moderate	Low	Moderate	Low	Low	Low	Moderate
Tilton, 2019 [28]	Moderate	Low	Low	Low	Low	Low	Low	Moderate

Risk of bias in randomized controlled trials assessed using Cochrane risk-of-bias tool for randomized trials (RoB2) [19]. Risk of bias in cohort studies assessed using Cochrane Risk of Bias in Non-Randomized Studies—of Interventions (ROBINS-I) [20].

## Data Availability

Not applicable.
